# 

**Published:** 1845-09

**Authors:** 


### TO SUBSCRIBERS.

[*Extract from the new Post Office Law.*]

"On and after July 1, 1845, on a letter not exceeding half an ounce in weight, sent any distance not exceeding three hundred miles, five cents.

"When sent any distance over three hundred miles, ten cents.

"For every additional weight of half an ounce, or any fractional excess of less than half an ounce, there shall be charged an additional postage of five or ten cents, according to the distance.

"On all pamphlets, magazines, periodicals, and every other kind and description of printed matter, (except newspapers, circulars, handbills and advertisements,) which shall be unconnected with any manuscript communication whatsoever, two and a half cents for every copy of no greater weight than one ounce for any distance. For every additional ounce, one cent; any fractional excess exceeding half an ounce, to be charged as an ounce; but any excess less than half an ounce is not to be regarded."

By the provisions of this Law it will be seen that Post Masters no longer have the power of remitting money to publishers free of postage; accordingly subscribers, when remitting the amount of subscription, will be mindful to enclose good current bank notes, and take care that the money and envelope do not weigh more than half an ounce; a sheet of letter paper, containing two bank notes, will come within this limit.

*The postage will be paid by the publishers on all letters containing money for subscriptions or advertisements*—in other instances it must be pre-paid.

In this proposal, subscribers to the Medical Examiner will see an additional evidence of a desire to regard their interests, and it is hoped they will be willing to reciprocate the disposition, by promptly complying with the terms of publication announced on the cover.

*July 1st*, 1845.

